# Supplementary material for: BRIE2: computational identification of splicing phenotypes from single-cell transcriptomic experiments
Source: Genome Biol. 2021 Aug 27;22:251. doi: 10.1186/s13059-021-02461-5 (PMC8393734; doi:10.1186/s13059-021-02461-5)
Supplement: Supplementary file 2 — Additional file 2 Review history. [file 13059_2021_2461_MOESM2_ESM.docx]

Review History

**First round of review**

**Reviewer 1**

**Were you able to assess all statistics in the manuscript, including the appropriateness of statistical tests used?**

Yes: The statistics in the manuscript are fine

**Were you able to directly test the methods?**

No

**Comments to author:**

In this paper, Huang and Sanguinetti describe BRIE2, a Bayesian model that predicts single-cell isoform usage from read information, sequence features, and cell-level covariates. This is an extension of the previous BRIE model published in Genome Biology—now the authors jointly model all cells at once and derive a variational approximation to avoid expensive MCMC sampling. The method is potentially interesting, but the relationship with existing methods is not clear, I do not understand the rationale for designing the model, and I have some questions about the method evaluation.

Major Comments:

1. What is the rationale for using sequence features to predict isoform usage? These sequences features are invariant (i.e., the same in every cell), assuming that all cells have the same genotype. Thus, I can't see how it makes sense to predict the variation in isoform usage across cells using sequence.

2. How does BRIE2 compare to notable existing methods for single-cell splicing analysis, such as Census (Qiu et al. Nature Methods 2017), Expedition (Botvinnik et al., Molecular Cell 2017), kallisto (Booeshaghi et al., bioRxiv 2020) and SCATS (Hu et al., PLoS Computational Biology 2020)? Each of these papers and methods had very similar goals to those in this paper, and it is important to explain how BRIE2 is different from and better than these methods. I am not immediately convinced of the novelty of this approach.

3. What is the advantage of BRIE2 compared to bulk analyses, such as running Cufflinks on all reads from each cluster in the single-cell dataset? For example, in the comparison of MS vs. control cells, it is not clear how the single-cell resolution adds anything to this analysis. As far as I can tell, the 368 differential splicing events could have been detected just as well using bulk RNA-seq and standard splicing analysis tools. Since this is a paper about single-cell splicing analysis, it is important to emphasize the unique insights one can glean from single-cell data, as opposed to traditional bulk analyses. For example, changes in population PSI variance or bimodality in isoform usage cannot be obtained from bulk data.

4. The results in Fig. 5 seem to show a qualitative improvement in velocity plots when genes are selected using BRIE2. However, it is important to establish this advantage using quantitative metrics.

5. The scvelo method provides a way to test for differential kinetics (likelihood ratio test) and subsequently adjust RNA velocity estimates to account for any differential kinetics. Does selecting differential momentum genes with BRIE2 still provide an advantage when the scvelo differential kinetic adjustment is performed? A related question: what cell covariates did you use when selecting differential momentum genes?

Minor Comments:

I found numerous typos throughout the methods section. I recommend proofreading this section more carefully. For example:

"mimics the log likelihood ratio in hypothesis test way"

"no any pre-processing is needed"

"for conventional reason, it refers"

"where we could use a deterministic way"

"evidence lower bounder"

**Reviewer 2**

**Were you able to assess all statistics in the manuscript, including the appropriateness of statistical tests used?**

No

**Were you able to directly test the methods?**

No

**Comments to author:**

Summary:

In this paper, the authors present an extension of the BRIE model to improve splicing quantification in scRNAseq data by incorporating cell level features. The BRIE2 model introduced here attempts to infer a posterior distribution of exon inclusion (PSI) by learning informative priors using a Bayesian linear model with sequence features (i.e. motifs, RBP binding sites, etc) and cell level information (i.e. cell type, disease state, etc) as regressors. The authors also propose using a model selection approach to compare models with and without cell type or disease state covariates as a means for differential splicing analysis. The BRIE2 model is evaluated on several synthetic datasets and is shown to produce posterior estimates that are highly concordant with true values, especially for lowly covered genes and to correctly select DS events (measured by AUC). BRIE2 is then applied to a multiple sclerosis dataset which produces PSI quantitation that are correlated to cell type. Finally, the authors use BRIE2 to select DS genes used in RNA velocity visualization and show that this approach recovers more accurate trajectories associated with known biology and a scNT-seq ground truth.

Overall, BRIE2 offers faster implementation and additional model features compared to BRIE. The paper also demonstrates potential usage of BRIE2 output for RNA velocity analysis. But as a paper introducing a method we found the manuscript to lack critical components which we discuss below.

Critique

=====

(1) The authors do not compare their method against any baseline models or alternative approaches. For example, even a simple baseline for splicing quantification such as a point estimate of PSI derived by dividing junction spanning reads by total reads.

(2) The paper makes claims about general modeling that include local sequence features (e.g. RBP binding sites) and cell features (e.g. cell type). However, there is no real evaluation of the former. This leaves only application to distinguish between cell groups, which (related to point #1 above) begs the question of what is the advantage of this approach compared to methods such as rMATS, MAJIQ, LeafCutter, etc. which are all fast, scalable and can capture many events (de-novo and complex) which BRIE2 is not able to capture.

(3) The authors should try to show how well a model without cell type information quantifies PSI compared to the model with this information as a function of coverage. In other words, when reads are sparse, how much of a gain is including cell type info?

(4) The experiments on multiple sclerosis don't show accuracy of PSI quantification. It simply shows that PSI correlates to cell type which will clearly happen when PSI is predicted from cell specific sequence features regardless of whether cell type information is included as a covariate. Thus this evaluation is insufficient. The PSI values could still separate cell types but be completely wrong.

(5) Why is Mbp selected? This gene have almost no fold change and this is reflected in the violin plot where all the groups have about the same mean.

(6) Although the authors show selection via BRIE2 DS seems to improve RNA velocity trajectory visualizations, is this selection process superior to, for example, simply using DE genes? We suspect using expression is sufficient due to correlation between expression changes and developmental trajectories.

(7) The method for computing the p-value for DS appears to be incorrect. The author's propose applying Wilk's theorem to a Bayes factor by claiming it is chi-squared distributed. The Bayes factor compares models with different numbers of parameters. A LRT (i.e. a t-test) has the same number of parameters under the null and alternative models. Wilk's theorem does not apply here. The authors should simply rank Bayes factors. For example, this is done in the scVI DE analysis.

(8) It is unclear why the authors choose to include ambiguous reads in their model. It is sufficient to use junction spanning reads and total reads to quantify PSI. Can the author show the addition of this variable leads to an improvement in model performance?

(9) The use of the logit model over PSI seems not appropriate. First, exon inclusion tends to be either high or low (i.e. 0 or 1). Under a logit model, density is concentrated at intermediate PSIs. Thus the predicted PSI distributions will not reflect true biology. Second, the resulting model is a Gaussian with a gene specific homeostatic error (Eq 3). This again seems not appropriate as it does not reflect variance in PSI space well and does not accommodate for dispersion as a function of coverage in the transformed space. It may be that the model still works extremely well and quantifies PSI and splicing changes extremely well, but as noted above that is not demonstrated in the manuscript.

(10) It is not clear why the simulation setup is realistic in any way. For example, why is \sigma set to 3? How is that reflecting real data?

(11) The exact nature of the sequence level features used as input could be made more clear.

Note:

=====

We didn't find included a code repository to test the method.

Typos:

=====

·	Page 1

o	50: identification of cell state

o	51: provide

·	Page 2

o	1: minimal

o	33: genomic sequences

o	37: run on sentence

·	Page 3:

o	For both -> both

·	Page 4:

o	50: cell type with well balance -> unclear what this means.

·	Page 6:

o	9: Observing this biological transition in more detail

·	Page 7:

o	7: conventional reasons.

o	8: Without loss of generality

o	48: In the BRIE2 model

**Reviewer 3**

**Were you able to assess all statistics in the manuscript, including the appropriateness of statistical tests used?**

Yes: It's a stats paper.

**Were you able to directly test the methods?**

No

**Comments to author:**

The authors extend their previous method BRIE in two ways. First, they make the prior on PSI depend on cell features (rather than just gene features as in BRIE1). Second, they switch out their previous Metropolis-Hastings + optimization for a stochastic variational approach. They present results on simulations, SMART-seq2 data from a mouse model of MS, and on two published RNA velocity examples. In the latter that choosing genes that are differentially spliced wrt to some relevant axis is beneficial.

The paper is reasonably clearly written in parts and has quite severe omissions in others. In particular:

1) what are the gene features used in each experiment? Presumably I could guess this from the BRIE1 paper but it should be explicit here.

2) The logic for using differentially spliced genes for RNA velocity is not clear at all. Intuitively I would have thought that differentially spliced genes would be BAD choices, since the relationship that scVelo (and related methods) want to leverage is that between unspliced and spliced mRNA as a proxy for transcription rate: differential splicing is a confounder in this context. However, I suspect what the authors may be doing is rather testing for differential "intron retention" rather than exon skipping. However, this is not really IR since the unspliced RNA is still expected to be spliced. If my understanding is correct, what they are actually doing is searching for differential _transcription rate_ (e.g. across cell types) and then using these genes in scVelo. This seems quite reasonable, but should be fully explained if so.

3) Even after reading the methods it is quite unclear to me how the ELBO is used to test for differential splicing. Is the full model retrained as described for each gene? This seems quite computationally intensive. Pseudocode might be a helpful way of describing the procedure more formally.

The paper is in need of careful reading over by an editor for grammatical mistakes.

My main technical concerns with the paper are

1. the "splicing PCs". It is interesting to see these are cell-type specific, but I worry about difference in total expression leaking in. In particular, as gene expression increases for a gene (with no change in psi), the counts will all increase. Ideally, this will not influence estimated psi, but there is no guarantee of this: the increased counts will increase the strength of the likelihood and pull the psi away from the prior mean and towards the empirical mean. The authors could address the magnitude of this effect by simulating counts with fixed psi and total expression varying according to a real dataset. Here no splicing PCs should be seen associating with cell type.

2. Lack of comparison to other approaches for differential splicing. There are other methods for single cell splicing analysis, and many methods for differential splicing. How does BRIE2 compare in terms of sensitivity/specificity?

The paper should make it clear whether BRIE2 can be used to assess differential splicing in 10x/3' tagged scRNAseq (not just for spliced vs unspliced).

Given that half the results presented are applied to RNA velocity which is really about transcription rate (despite using spliced vs unspliced reads), the title/abstract/introduction should probably be reworked to better incorporate this aspect of the work.

Minor: the methods should refer to a "splicing gene" as a splicing event. There could be multiple events per gene.

I've highlighted some typos on the manuscript added.

**Authors Response**

**Point-by-point responses to the reviewers’ comments:**

*We thank you and the reviewers for the detailed and constructive feedback. According to the reviewers' comments, we have comprehensively re-written the manuscript to add more explanations and additional experimental validations. The major changes to the manuscript in this revivision are as follows:*

*1.	We have added comparison to existing methods (rMATS and MAJIQ) for differential splicing detection with newly simulated scRNA-seq data. These results are shown in the new main Figure 2.*

*2.	We have clarified the ELBO-gain as an approximation of Bayes factor for model selection, and removed its link to likelihood ratio test.*

*3.	We have clarified the motivation for using differential momentum genes in RNA-velocity studies, and added a table quantifying the benefits of doing so (Supp. Table S1).*

*4.	We have added a table describing the usage modes of BRIE2 in the new Table 1*

*(with gene, cell or none features) and their according purpose and benefits.*

*You will find our point-by-point response to the reviews below. We hope that you agree with us that all the concerns have been well addressed and this manuscript is ready for publication in Genome Biology.*

*We look forward to hearing back from you soon.*

*Yours sincerely,*

*Yuanhua Huang & Guido Sanguinetti*

*University of Hong Kong*

*SISSA, Trieste*

*University of Edinburgh*

*Point-by-point response*

*Reviewer 1: page 2-4*

*Reviewer 2: page 5-8*

*Reviewer 3: page 9-11*

Reviewer #1:

In this paper, Huang and Sanguinetti describe BRIE2, a Bayesian model that predicts single-cell isoform usage from read information, sequence features, and cell-level covariates. This is an extension of the previous BRIE model published in Genome Biology—now the authors jointly model all cells at once and derive a variational approximation to avoid expensive MCMC sampling. The method is potentially interesting, but the relationship with existing methods is not clear, I do not understand the rationale for designing the model, and I have some questions about the method evaluation.

Major Comments:

1.	What is the rationale for using sequence features to predict isoform usage? These sequences features are invariant (i.e., the same in every cell), assuming that all cells have the same genotype. Thus, I can't see how it makes sense to predict the variation in isoform usage across cells using sequence.

*Response: We do not use sequence features for testing for differential isoform usage, we apologise for the confusion. The sequence features were introduced in BRIE v1 (Genome Biology, 2017), where we aimed to use the sequence features to learn an informative prior on PSI. Importantly BRIE1 is normally used independently on each cell, so that different cells might have different sequence-informed priors (for example reflecting different importance of DNA-binding proteins in different cells). It is however true that these gene features are indeed invariant across cells, and the learned prior is generally similar across most cells, while minor differences still exist due to the different fitted weights alpha (cell-by-sequence_features). Therefore, it provides a way to share information from highly covered genes to lowly covered genes through a regularization from sequence features. This strategy, proposed in BRIE1, hence balances the cell “invariant” imputation and cell observed reads in a coherent way, and can consequently enhance the downstream analysis, e.g., different splicing detection. Additionally, the sequence features can be used to model the genotype changes between different cells (or across individuals).*

*For BRIE2, while the framework retains the support of either gene or cell or both types of features, the focus shifts from quantification of PSI to detection of variable splicing events associating with given covariates, e.g., cell type or pseudo time. Hence, the gene sequence features are an optional argument. In this revision, we included an extensive discussion of this topic at the start of the Results section (page 2-4) as well as a new table (Table 1) to highlight the different purposes of the settings of BRIE2.*

2.	How does BRIE2 compare to notable existing methods for single-cell splicing analysis, such as Census (Qiu et al. Nature Methods 2017), Expedition (Botvinnik et al., Molecular Cell 2017), kallisto (Booeshaghi et al., bioRxiv 2020) and SCATS (Hu et al., PLoS Computational Biology 2020)? Each of these papers and methods had very similar goals to those in this paper, and it is important to explain how BRIE2 is different from and better than these methods. I am not immediately convinced of the novelty of this approach.

*Response: We agree with the reviewer that it is indeed important to benchmark with other existing methods, an issue that has been highlighted also by Reviewer 2. As mentioned BRIE2 has two components: quantification of PSI and detection of variable splicing events. For the quantification accuracy, we have compared BRIE2 with BRIE1 on real data and evaluate BRIE2 on simulated data (Supp. Figs. S4 and S5), confirming its strong accuracy. BRIE1 was also compared to Kallisto, Census and Cufflinks and found to be more accurate in the original BRIE1 paper. We now explicitly mention these earlier comparisons on page 4. For Expedition, the focus is on visualization and categorising splicing events according to modality (whether a single isoform or more are expressed in a given cell), instead of detection of variable splicing events, hence not directly comparable for BRIE2. Additionally, Expedition has very high coverage requirements (the original paper obtained several million reads per cell, which is not comparable with standard smart-seq2 data sets).*

*Concerning differential quantification, which is the main focus of this paper ,we followed Reviewer 2’s suggestion and added comparison with rMATS and MAJIQ, both of which are able to detect differential splicing, though developed for bulk RNA-seq .As shown in the new main Fig. 2 and Supp. Fig. S6-7, both rMATS and MAJIQ are effective at detecting differential events, but BRIE2 clearly outperforms them both by a substantial margin, in particular for groups of cells exhibiting subtle to moderate differences. Additionally, BRIE2 is the only method that can detect variable splicing events associated with continuous covariates, e.g., pseudotime and drug doses.*

*We also attempted to use SCATS to provide an additional relevant for comparison. However, when running SCATS, we obtained substantially lower accuracies as well as very slow execution times. We have contacted the authors who confirmed that situations with very low unambiguous read counts (which is the focus of BRIE2) can give rise to numerical problems in SCATS. The results of these experiments are shown in Fig. R1 below (related to the new main Fig. 2), however, given the issues with SCATS, we chose not to include these results in the main manuscript.*

*Figure R1. The ROC curve and Precision-recall curve for evaluating SCATS in detecting differential alternative splicing events. The simulation settings and effect size level L2 to L5 are the same as main Fig. 2.*

3.	What is the advantage of BRIE2 compared to bulk analyses, such as running Cufflinks on all reads from each cluster in the single-cell dataset? For example, in the comparison of MS vs. control cells, it is not clear how the single-cell resolution adds anything to this analysis. As far as I can tell, the 368 differential splicing events could have been detected just as well using bulk RNA-seq and standard splicing analysis tools. Since this is a paper about single-cell splicing analysis, it is important to emphasize the unique insights one can glean from single-cell data, as opposed to traditional bulk analyses. For example, changes in population PSI variance or bimodality in isoform usage cannot be obtained from bulk data.

*Response: The main reason for splicing analysis in single cells is to quantify the PSI value at each cell, which not only gives the mean, but also the full distribution in the cell population, which BRIE2 can retain by quantifying PSI for each cell. In bulk samples, one can only detect the averaged effect on differential splicing, while using BRIE2 with single cells, we can also account for the variability. Consequently, BRIE2 strongly outperforms state-of-the-art bulk methods such as rMATS and MAJIQ, as noted earlier. Additionally, BRIE2 also supports continuous cell variables, e.g., development time and can remove confounder covariates, which cannot be captured by conventional bulk analysis. We also followed the reviewer’s suggestion and added a number of example genes in Supp. Fig. S11 (discussed in the text on pages 7-8), clearly showing the non-trivial distribution of splicing ratios across the populations, including bimodal and long-tailed behaviours.*

4.	The results in Fig. 5 seem to show a qualitative improvement in velocity plots when genes are selected using BRIE2. However, it is important to establish this advantage using quantitative metrics.

*Response: We agreed that quantitative metrics are important for the comparison, hence added a score of cross-boundary correctness, as shown in Methods (page 14) and the new Supp. Table S1.*

5.	The scvelo method provides a way to test for differential kinetics (likelihood ratio test) and subsequently adjust RNA velocity estimates to account for any differential kinetics. Does selecting differential momentum genes with BRIE2 still provide an advantage when the scvelo differential kinetic adjustment is performed? A related question: what cell covariates did you use when selecting differential momentum genes?

*Response: Thanks for the very relevant suggestions. The differential kinetics aims to test whether the kinetic rates are different in one group of cells versus all others, which may provide a list of dynamic related genes. However, from our observations, the differential kinetic genes both between cell types (new Supp. Fig. S12b-d) or between time points (new Supp. Fig. S18b-d) did not help in identifying the cell transition trajectories. The cell covariates that were used were the cell type for the Dentate Gyrus data set and the stimulation time for the scNT data set, as explained now in the main text.*

Minor Comments:

I found numerous typos throughout the methods section. I recommend proofreading this

section more carefully. For example:

"mimics the log likelihood ratio in hypothesis test way"

"no any pre-processing is needed"

"for conventional reason, it refers"

"where we could use a deterministic way"

"evidence lower bounder"

*Response: All corrected with thanks.*

Reviewer #2:

In this paper, the authors present an extension of the BRIE model to improve splicing quantification in scRNAseq data by incorporating cell level features. The BRIE2 model introduced here attempts to infer a posterior distribution of exon inclusion (PSI) by learning informative priors using a Bayesian linear model with sequence features (i.e. motifs, RBP binding sites, etc) and cell level information (i.e. cell type, disease state, etc) as regressors. The authors also propose using a model selection approach to compare models with and without cell type or disease state covariates as a means for differential splicing analysis. The BRIE2 model is evaluated on several synthetic datasets and is shown to produce posterior estimates that are highly concordant with true values, especially for lowly covered genes and to correctly select DS events (measured by AUC). BRIE2 is then applied to a multiple sclerosis dataset which produces PSI quantitation that are correlated to cell type.

Finally, the authors use BRIE2 to select DS genes used in RNA velocity visualization and show that this approach recovers more accurate trajectories associated with known biology and a scNT-seq ground truth.

Overall, BRIE2 offers faster implementation and additional model features compared to BRIE. The paper also demonstrates potential usage of BRIE2 output for RNA velocity analysis. But as a paper introducing a method we found the manuscript to lack critical components which we discuss below.

Critique

(1)	The authors do not compare their method against any baseline models or alternative approaches. For example, even a simple baseline for splicing quantification such as a point estimate of PSI derived by dividing junction spanning reads by total reads.

*Response: It is indeed important to compare to other existing methods. For the quantification accuracy, we compared BRIE2 with BRIE1 on experimental data and found they are highly consistent (the Supp. Fig. S4). We stress that BRIE1 was extensively benchmarked against a number of strong competitors in the original paper (YH and GS, Genome Biology 2017). In this manuscript, we have a focus on evaluating differential splicing detection, and compared to rMATS and MAJIQ in this revision (see the response to the next question).*

(2)	The paper makes claims about general modeling that include local sequence features (e.g. RBP binding sites) and cell features (e.g. cell type). However, there is no real evaluation of the former. This leaves only application to distinguish between cell groups, which (related to point #1 above) begs the question of what is the advantage of this approach compared to methods such as rMATS, MAJIQ, LeafCutter, etc. which are all fast, scalable and can capture many events (de-novo and complex) which BRIE2 is not able to capture.

*Response: As mentioned in the response to Reviewer 1, the evaluation of the effect of local sequence features was the main focus of the BRIE1 paper, which indeed has a comprehensive comparison with other methods for PSI quantification. In this manuscript, we focus on the problem of detecting variable splicing events in a cell population, e.g., between cell types or along pseudotime. We agree with the reviewer that a more thorough comparison with existing methods (developed for bulk RNA-seq) would be an appropriate addition, to do so we further extended our simulation to generate read sequences, so as to run these counterparts (rMATS and MAJIQ). As shown in the new main Fig 2 and Supp. Fig. S6-7, BRIE2 clearly outperforms all these two methods in detecting differential splicing events between two groups of cells for subtle to large differences. This is thanks to BRIE2’s capability of modelling the uncertainty of PSI quantification. Additionally, BRIE2 is the only method that can detect variable splicing events associated with continuous covariates, e.g., pseudotime and drug doses. We acknowledge however that, unlike rMATS and MAJIQ, BRIE2 cannot handle complex / de-novo events, although we would be very surprised if any bulk method were able to reliably call complex/ de-novo events in single cell data due to the high levels of sparsity of scRNA-seq data sets. We have added a final paragraph to this effect to the Conclusion section (page 9).*

(3)	The authors should try to show how well a model without cell type information quantifies PSI compared to the model with this information as a function of coverage. In other words, when reads are sparse, how much of a gain is including cell type info?

*Response: Thanks for this comment. In the new Supp. Fig. S5, we compared the quantification accuracy of Psi on simulated data by using or not using the cell feature which is a unity feature for aggregated prior (mode 2-quant vs mode 0). Using coverage levels from a real scRNA-seq data set, the usage of a single cell-level feature improved the Pearson correlation coefficient (PCC) between the ground truth and the inferred PSI from 0.781 to 0.960(panels a-b). We further quantified the improvement in PCC at different coverage levels (panels c-d), as suggested by the reviewer, showing a very substantial improvement in quantification of PSI by using a cell-level feature at low coverage (median number of reads per event below 20).*

(4)	The experiments on multiple sclerosis don't show accuracy of PSI quantification. It simply shows that PSI correlates to cell type which will clearly happen when PSI is predicted from cell specific sequence features regardless of whether cell type information is included as a covariate. Thus this evaluation is insufficient. The PSI values could still separate cell types but be completely wrong.

*Response: We thank the reviewer for raising this issue. However, as an experimental dataset, this is a lack of ground truth of the PSI value, hence it is generally impossible to evaluate the accuracy of PSI quantification. One thing we’d like to clarify is that the cell type information is not included in the initial PSI quantification (see the revised text in page 5). Still, the PSI is highly predictive of cell type and disease condition, which are indirect evidence on the informativeness of PSI quantification.*

(5)	Why is Mbp selected? This gene have almost no fold change and this is reflected in the violin plot where all the groups have about the same mean.

*Response: We chose this gene because there is a subtle but significant difference between MS and control in MOL cell type (median: 0.1 vs 0.15), which is in accordance with known biology of the disease (as pointed out in the original data paper). In this revision, we have included additional 6 most significant genes in Supp. Fig. S11.*

(6)	Although the authors show selection via BRIE2 DS seems to improve RNA velocity trajectory visualizations, is this selection process superior to, for example, simply using DE genes? We suspect using expression is sufficient due to correlation between expression changes and developmental trajectories.

*Response: We thank the reviewer for suggesting this relevant alternative. To fully answer this question, we need a quantitative assessment of improvement in trajectory visualisation; as suggested by Reviewer 3, we used cross-boundary direction correctness (added in Methods, page 14) to provide such quantification. We then used edgeR to perform DE-based gene selection (using the same design matrix as BRIE2) and found the trajectory based on DE genes are less consistent with the expected direction compared to BRIE2’s DM genes (Supp. Table S1; Supp. Fig. S12a and S18a).*

(7)	The method for computing the p-value for DS appears to be incorrect. The author's propose applying Wilk's theorem to a Bayes factor by claiming it is chi-squared distributed. The Bayes factor compares models with different numbers of parameters. A LRT (i.e. a t-test) has the same number of parameters under the null and alternative models. Wilk's theorem does not apply here. The authors should simply rank Bayes factors. For example, this is done in the scVI DE analysis.

*Response: We very much appreciate the reviewer’s comments on this, which correctly picks up a sleight of hand on our part. Indeed, we didn’t prove that the ELBO gain follows the chi-squared distribution, but rather showed that it calibrated well with the LRT under the null hypothesis. We have taken the suggestion from the reviewer and removed the link between ELBO gain and LRT. Instead, we further clarified that the ELBO gain acts as an approximate Bayes factor and can be directly used to select DAS events or DMG.*

(8)	It is unclear why the authors choose to include ambiguous reads in their model. It is sufficient to use junction spanning reads and total reads to quantify PSI. Can the author show the addition of this variable leads to an improvement in model performance?

*Response: we thank the reviewer for the good question. In the new Supp. Fig. S3, we show the difference between using and not using ambiguous reads in quantifying PSI. Briefly, the ambiguous reads function as additional normalization, compared to only using the unambiguous reads. This can be particularly useful in cases where one isoform is under-represented by unambiguous reads due to its short length (i.e., short isoforms tend to have very few unambiguous reads simply because the length of the unambiguous region is very short, which leads to underestimate their abundance).*

(9)	The use of the logit model over PSI seems not appropriate. First, exon inclusion tends to be either high or low (i.e. 0 or 1). Under a logit model, density is concentrated at intermediate PSIs. Thus the predicted PSI distributions will not reflect true biology. Second, the resulting model is a Gaussian with a gene specific homeostatic error (Eq 3). This again seems not appropriate as it does not reflect variance in PSI space well and does not accommodate for dispersion as a function of coverage in the transformed space. It may be that the model still works extremely well and quantifies PSI and splicing changes extremely well, but as noted above that is not demonstrated in the manuscript.

*Response: Thanks for the comments. In fact, a logit-normal model can well present a U-shape distribution of PSI values, e.g., when mean=0 and sigma=5. Indeed, as shown in Eq3, each gene has its own sigma in the logit-normal distribution. Actually, this setting is practically useful because it has enough flexibility to allow each gene to have a different distribution, particularly in terms of dispersion. Note, Eq. 3 is the prior distribution, which means all cells share this prior and its dispersion reflects that of the cell population, and may not need to account for coverage. On the other hand, the variational posterior (Eq. 5) has a sigma for each gene and each cell, which is flexible enough to reflect coverage and other factors. We would also like to point to a recent paper https://doi.org/10.7554/eLife.54603 demonstrating that the 0/1 nature of excon inclusion might be a coverage-dependent artefact, suggesting that hardwiring such behaviour in a prior distribution might be inappropriate.*

(10)	It is not clear why the simulation setup is realistic in any way. For example, why is \sigma set to 3? How is that reflecting real data?

*Response: thank you for the comment on checking the choice of the simulation parameters. We completely re-designed the simulation (see Methods section), directly simulating reads (as opposed to just counts) and using seed (real) data to learn parameters for realistic simulations. As a result, a major difference is that we changed the mean of z from 0, (a fixed value; Psi=0.5), to the observed mean from seed data, and also changed the \sigma from 3 to 1, as it was the mode in the observations (new Supp. Fig. S22). We hope this revised simulation setting is better representing the real observations in experiments.*

(11)	The exact nature of the sequence level features used as input could be made more clear.

*Response: Agreed, but this is because we do not evaluate the sequence level features in this paper, they are included as a legacy from BRIE1 in the software. We have clarified this point (and referred to the original BRIE1 publications for sequence level modelling) in the first section of results (page 4; together with the newly added Table 1).*

Note:

We didn't find included a code repository to test the method.

*Response: They are all listed in the original manual. Now, we have moved these links to the “Availability of Data and Materials” section for easier navigation.*

Typos:

·	Page 1

o	50: identification of cell state

o	51: provide

·	Page 2

o	1: minimal

o	33: genomic sequences

o	37: run on sentence

·	Page 3:

o	For both -> both

·	Page 4:

o	50: cell type with well balance -> unclear what this means.

·	Page 6:

o	9: Observing this biological transition in more detail

·	Page 7:

o	7: conventional reasons.

o	8: Without loss of generality

o	48: In the BRIE2 model

*Response: all fixed with thanks.*

Reviewer #3:

The authors extend their previous method BRIE in two ways. First, they make the prior on PSI depend on cell features (rather than just gene features as in BRIE1). Second, they switch out their previous Metropolis-Hastings + optimization for a stochastic variational approach. They present results on simulations, SMART-seq2 data from a mouse model of MS, and on two published RNA velocity examples. In the latter that choosing genes that are differentially spliced wrt to some relevant axis is beneficial.

The paper is reasonably clearly written in parts and has quite severe omissions in others. In particular:

1)	what are the gene features used in each experiment? Presumably I could guess this from the BRIE1 paper but it should be explicit here.

*Response: We thank the reviewer for highlighting this issue, also pointed out by Rev 2. This is because we do not evaluate the sequence level features in this paper, they are included as a legacy from BRIE1 in the software. We have clarified this point (and referred to the original BRIE1 publications for sequence level modelling) in the first section of results (page 4; together with the newly added Table 1).*

2)	The logic for using differentially spliced genes for RNA velocity is not clear at all. Intuitively I would have thought that differentially spliced genes would be BAD choices, since the relationship that scVelo (and related methods) want to leverage is that between unspliced and spliced mRNA as a proxy for transcription rate: differential splicing is a confounder in this context. However, I suspect what the authors may be doing is rather testing for differential "intron retention" rather than exon skipping. However, this is not really IR since the unspliced RNA is still expected to be spliced. If my understanding is correct, what they are actually doing is searching for differential _transcription rate_ (e.g. across cell types) and then using these genes in scVelo. This seems quite reasonable, but should be fully explained if so.

*Response: we thank the reviewer for pointing out this difficulty, and for the effort of unravelling our admittedly rather convoluted logic! Yes, the line of thought is the following: feature selection is an important preprocessing step for RNA velocity analyses (see also ref 23 in the new manuscript), and it stands to reason that genes with differential transcription rate should be the most informative. Differential transcription rate can manifest itself with a change in the proportion of unspliced reads (this is in fact the main RNA velocity argument), leading to changes in apparent intron retention (although the reviewer is right that these reads will eventually be spliced, so it is purely an apparent effect). Intron retention formally can be treated as a type of alternative splicing, and this is what we do with BRIE2. We have now discussed more clearly this line of thought at the beginning of the relevant section (pages 9-10; and also background in page 2).*

3)	Even after reading the methods it is quite unclear to me how the ELBO is used to test for differential splicing. Is the full model retrained as described for each gene? This seems quite computationally intensive. Pseudocode might be a helpful way of describing the procedure more formally.

*Response: We thank the reviewer for raising this issue, we agree that the wording was poor, partly due to our attempt to explain the BRIE2 model in full generality. We have now described more in detail the various modes of usage of BRIE2 (see new Table 1), with the relevant mode for differential testing being Mode 2-diff. The important point to notice is that, when testing for differential splicing, the gene-level features are irrelevant (assuming genes in the two conditions have the same sequence). So we only use BRIE2 in Mode 2-diff (with cell-level features but without gene level features), which means all events are independent. Therefore, both model M0 (no effect) and M1 (significant effect) are trained once for all events simultaneously, which only incurs a computational cost of twice the original cost of training a BRIE2 model. We have added this explanation to the Methods section page 12 (Eq. 9 and Eq. 10), where we also point out the restriction that all cells should share the same sequence for this procedure to hold exactly.*

The paper is in need of careful reading over by an editor for grammatical mistakes.

*Response: we thank the reviewer for this kind suggestion and have made a substantial overhaul of the text in this revision.*

My main technical concerns with the paper are

1.	the "splicing PCs". It is interesting to see these are cell-type specific, but I worry about difference in total expression leaking in. In particular, as gene expression increases for a gene (with no change in psi), the counts will all increase. Ideally, this will not influence estimated psi, but there is no guarantee of this: the increased counts will increase the strength of the likelihood and pull the psi away from the prior mean and towards the empirical mean. The authors could address the magnitude of this effect by simulating counts with fixed psi and total expression varying according to a real dataset. Here no splicing PCs should be seen associating with cell type.

*Response: we much appreciated this technical comment. Indeed, this was our initial concern too. However, we expect that this effect can be mitigated by using an aggregation based prior through adding a unity cell feature (namely an intercept for each gene; Mode 2-quant in Table 1). In that case, for lowly covered cells, the Psi will be close to the prior, i.e., the (adapted) mean in the population. Therefore, the Psi PC will primarily present the splicing events with genuine variability and sufficient reads support. This argument is well supported by the newly added simulation as suggested by the reviewer (Supp. Fig. S8). When we set the same Psi vector for all cells, and keep the same expression level to the real data, we found that the estimated Psi PCs (top three) do not show any variation across cell types.*

2.	Lack of comparison to other approaches for differential splicing. There are other methods for single cell splicing analysis, and many methods for differential splicing. How does BRIE2 compare in terms of sensitivity/specificity?

*Response: As also mentioned in the response to the other reviewers, we have added comparison to rMATS and MAJIQ and shows superior performance of BRIE2 in Fig 2 and Supp. Fig. S6-7 and relevant sections. We have also compared to SCATS, which is designed as a single-cell method, but unfortunately that method encountered numerical issues that could not be resolved even after discussions with the authors (see Fig R1 in response to Reviewer 1).*

The paper should make it clear whether BRIE2 can be used to assess differential splicing in 10x/3' tagged scRNAseq (not just for spliced vs unspliced).

*Response: Thanks for pointing out. At the current stage, BRIE2 is not applicable for 10x/3’ data, but its framework is readily applicable for variable splicing detection if the isoform read count matrices, e.g., for SE or alternative poly-A or alternative TSS, can be obtained from other tools. We added the discussion in the Conclusion section (page 9).*

Given that half the results presented are applied to RNA velocity which is really about transcription rate (despite using spliced vs unspliced reads), the title/abstract/introduction should probably be reworked to better incorporate this aspect of the work.

*Response: Thanks for this good comment. We have adapted our abstract and introduction for this purpose.*

Minor: the methods should refer to a "splicing gene" as a splicing event. There could be multiple events per gene.

*Response: good point. We have changed the splicing gene to splicing events, and specified if using interchangeably.*

I've highlighted some typos on the manuscript added.

*Response: Noted and corrected with many thanks.*

**Second round of review**

**Reviewer 3**

The authors have done a nice job addressing my comments/concerns and those of the other reviewers. I recommend acceptance.
